# Comparative analysis of sterol acquisition in the oomycetes *Saprolegnia parasitica* and *Phytophthora infestans*

**DOI:** 10.1371/journal.pone.0170873

**Published:** 2017-02-02

**Authors:** Paul Dahlin, Vaibhav Srivastava, Sophia Ekengren, Lauren S. McKee, Vincent Bulone

**Affiliations:** 1 Division of Glycoscience, School of Biotechnology, Royal Institute of Technology (KTH), AlbaNova University Centre, Stockholm, Sweden; 2 Department of Ecology, Environment and Plant Sciences, Stockholm University (SU), Stockholm, Sweden; 3 ARC Centre of Excellence in Plant Cell Walls and School of Agriculture, Food and Wine, The University of Adelaide, Waite Campus, Urrbrae, Australia; Agriculture and Agri-Food Canada, CANADA

## Abstract

The oomycete class includes pathogens of animals and plants which are responsible for some of the most significant global losses in agriculture and aquaculture. There is a need to replace traditional chemical means of controlling oomycete growth with more targeted approaches, and the inhibition of sterol synthesis is one promising area. To better direct these efforts, we have studied sterol acquisition in two model organisms: the sterol-autotrophic *Saprolegnia parasitica*, and the sterol-heterotrophic *Phytophthora infestans*. We first present a comprehensive reconstruction of a likely sterol synthesis pathway for *S*. *parasitica*, causative agent of the disease saprolegniasis in fish. This pathway shows multiple potential routes of sterol synthesis, and draws on several avenues of new evidence: bioinformatic mining for genes with sterol-related functions, expression analysis of these genes, and analysis of the sterol profiles in mycelium grown in different media. Additionally, we explore the extent to which *P*. *infestans*, which causes the late blight in potato, can modify exogenously provided sterols. We consider whether the two very different approaches to sterol acquisition taken by these pathogens represent any specific survival advantages or potential drug targets.

## Introduction

The sterols are a highly diverse group of isoprenoid-derived amphipathic biomolecules which play important structural and physical roles in all eukaryotic cells [1–6]. The precursors to sterol synthesis are isopentenyl diphosphate (IPP) and dimethylallyl pyrophosphate (DMAPP), which arise via the mevalonate (MVA) pathway, or alternatively via the methylerythritol phosphate (MEP) pathway in green algae and some red algae [7, 8].

**Fig 1:**
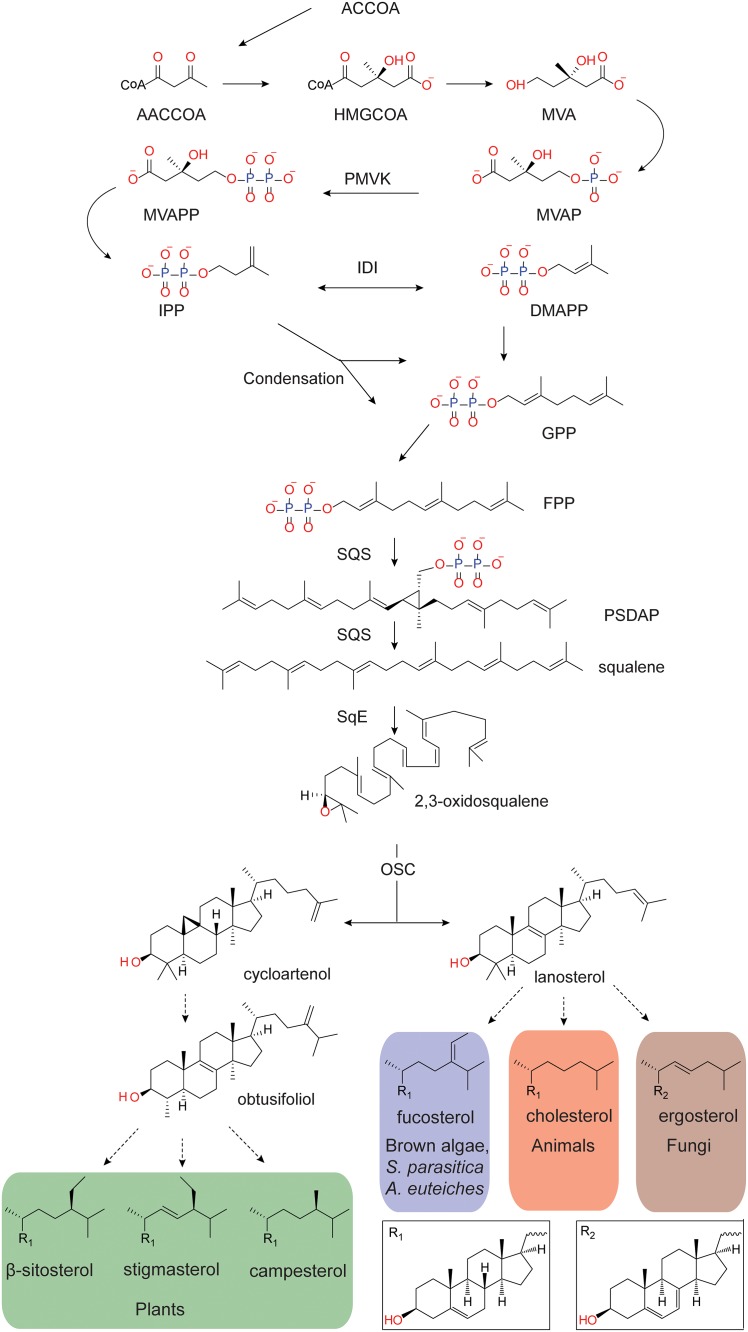
Generalised sterol biosynthesis pathway. Dashed arrows indicate that multiple enzymatic steps are occurring. The end sterols β-sitosterol, stigmasterol, campesterol, fucosterol and cholesterol have a common sterol nucleus structure (R_1_, shown in the box) differing only at the side chain, while the core of ergosterol (R_2_, shown in the box) has an additional point of desaturation in the sterol core.

Committed sterol biosynthesis begins with the production and subsequent epoxidation of squalene by respectively a squalene synthase (SQS) and a squalene epoxidase (SqE) (Fig 1) [6, 9]. The resulting 2,3-oxidosqualene is then cyclised by an oxidosqualene cyclase (OSC). Depending on the organism this reaction will produce either lanosterol or cycloartenol (Fig 1). These are then modified in a series of enzymatic steps (Fig 1). Oxidosqualene cyclisation is the last step in the pathway which is conserved in all sterol synthesising organisms (Fig 1) [10]. This divergence in the main pathway, and all the potential subsequent modifications, means that the final profile of sterols differs significantly between organisms (Fig 1).

The oomycetes are a diverse group of filamentous eukaryotic microorganisms, which includes important animal and plant pathogens. The oomycetes share many characteristic features with fungi, but are phylogenetically distinct from them and are grouped within the Stramenopiles (Heterokonta) phylum. Oomycete species differ greatly in their requirements for sterol provision [11–17]. Species of the Saprolegniales order are able to grow on sterol-free media, and possess genes with predicted roles in sterol biosynthesis [12, 14, 16–19]. Conversely, members of the Peronosporales order such as *Phytophthora* species are unable to synthesise sterols *de novo* due to an inability to produce oxidosqualene (Fig 1) [16, 17], as they lack conventional SqE genes [20].

Insects and nematodes also lack the capacity to synthesise oxidosqualene [21–25], but some are able to modify exogenous sterols [25, 26]. Intriguingly, a gene encoding a putative Δ^7^ sterol reductase has been reported in a *Phytophthora* species [12], suggesting a similar potential for sterol modification. Indeed, limited modification of sterols has been observed in *Phytophthora* species [27, 28]. Nonetheless, the profile of host sterols is important in determining the success of Peronosporales oomycetes, highlighted by the impaired growth of *Plasmopara viticola* on grapevine with inhibited sterol synthesis [29].

The extent and importance of sterol synthesis in the oomycetes is a matter of ongoing debate [14–16, 28, 30, 31], and the potential for sterol modification in Peronosporales species [12] adds further uncertainty. A clear understanding of the requirements and tolerance of pathogenic oomycetes for sterols is important for the development of effective pest management strategies. Here we present our assessment of the sterol requirements for growth in *S*. *parasitica* and *P*. *infestans*. We focus particularly on the ability of *P*. *infestans* to take up and modify exogenously provided sterols, and of *S*. *parasitica* to modulate its sterol profile in different growth media. In addition to a comparative sterol profile analysis of the two species, we have analysed the expression of multiple genes with predicted functions in sterol biosynthesis, including two *P*. *infestans* genes putatively encoding sterol modifying enzymes. From these data, we are able to reconstruct an entire *S*. *parasitica* sterol synthesis pathway, identifying multiple potential routes of biosynthesis and an additional gene which was not apparent in previous investigations [18, 19]. This builds on our recent biochemical demonstration that *S*. *parasitica* sterol biosynthesis begins with lanosterol production [32]. We could find no evidence that *P*. *infestans* is capable of sterol modification, and discuss how this compares with the approach taken by *S*. *parasitica*.

## Materials and methods

### Chemicals and reagents

Squalene, cholesterol, β-sitosterol, lanosterol, cycloartenol, campesterol, lathosterol, desmosterol, fucosterol, phosphatidylinositol, phosphatidylcholine, phosphatidylethanolamine, phosphatidylserine, phosphatidic acid, lysophosphatidylethanolamine, lysophosphatidylcholine and ceramide phosphorylethanolamine were purchased from Sigma Aldrich (St Louis, MO, USA). Zymosterol was purchased from Avanti Polar Lipids (Alabaster, AL, USA) and ergosterol was purchased from Supelco (Sigma-Aldrich Sweden AB, Stockholm, Sweden). All other growth media components were purchased from Sigma Aldrich (St Louis, MO, USA) and were of microbiological grade.

### Culturing of *Saprolegnia parasitica* and *Phytophthora infestans*

Strains of *P*. *infestans* (Mont.) de Bary 1876 (CBS 120920; GenBank JX418021) and *S*. *parasitica* Coker 1923 (CBS 223.65; GenBank JX418013) were obtained from the Centraal Bureau voor Schimmel Culture (CBS, Baarn, The Netherlands). Cultivars were separately maintained in artificial media. *S*. *parasitica* was maintained on defined Machlis medium [33] supplemented with 2% plant agar (Duchefa Biochemie) at 20°C. *P*. *infestans* was maintained on defined l-asparagine medium supplemented with 2% agar [34]. For sterol analysis of *S*. *parasitica*, an agar plug of 8 mm diameter with *S*. *parasitica* mycelium was excised with a cork borer and used to inoculate yeast mold (YM) liquid medium, Peptone liquid medium, or Machlis liquid medium [33]. The YM medium was prepared after Warrilow *et al*. [19]. The Peptone media was prepared with 6% glucose and 3% peptone in tap water. *P*. *infestans* mycelium for sterol analysis was cultured in defined l-asparagine medium [34] and inoculated by excising an 8 mm agar plug from the stock culture.

### Sterol analysis by GC-MS

#### Extraction of sterols from oomycete mycelium

Lipid fractions were extracted from mycelium using the method of Bligh and Dyer [35]. Mycelium was homogenised under liquid nitrogen to a fine powder. 3.75 volumes of chloroform:methanol (1:2) were added to 1 volume of sample. The mixture was incubated for 20 min at 60°C and centrifuged at 450 *g* for 10 min, at 17°C. The supernatant fluid was retained and the pellet resuspended in 3.75 volumes of chloroform:methanol (1:2). The mixture was again incubated for 20 min at 60°C and centrifuged at 450 *g* for 10 min, at 17°C. The supernatant fluid was washed by adding 2.5 volumes of water and 2.5 volumes of chloroform, followed by homogenisation by vortexing. The mixture was subsequently centrifuged at 17°C at 450 *g* for 10 min. The chloroform phase, containing the lipid extract, was recovered and dried under nitrogen gas. Nitrogen-dried lipid samples were resuspended in 3 mL hexane. Free sterols were separated from esterified sterols and other lipids on an SPE column (# 4600050c, ISOLUTE^®^ Si, 500MG/6ML) as described previously [36]. Esterified sterols were dried under nitrogen gas and saponified by adding 1 mL 2M KOH (in 95% ethanol) and kept for 45 min at 60°C. The alkaline hydrolysis was stopped by cooling down the tubes in cold water and adding 1 mL of H_2_O, 2 mL hexane, and 0.1 mL ethanol, and shaking vigorously. After centrifugation at 450 *g* for 10 min at 17°C, the upper hexane phase was transferred into small glass tubes and the remaining unsaponifiable compounds were dried under nitrogen gas for derivatisation.

#### Extraction of sterols from Machlis medium, YM medium, Peptone medium, and separate medium components

Aliquots of growth medium were taken and 3.75 volumes of chloroform:methanol (1:2) added prior to incubation at 60°C for 20 min. Five volumes of chloroform were then added and the sample was sonicated for 20 min in a water bath at 20°C. The mixture was centrifuged at 17°C at 450 *g* for 10 min and the supernatant fluid with the lipid fraction was saved. Nitrogen-dried lipid samples were resuspended in 3 mL hexane and processed as described above. The sterol extraction method for the media components yeast mold and peptone was performed as described for homogenised mycelia.

#### GC-MS analysis

Free sterols and unsaponifiable sterols were derivatised by adding bis(trimethylsilyl)trifluoroacetamide (BSTFA) and trimethylchlorosilane (TMCS), 99:1 (#33155 Supelco, Sigma-Aldrich Sweden AB, Stockholm, Sweden), with pyridine (1/1 v/v) and incubating at 60°C for 1 hr. Silylated products were dried under nitrogen gas and diluted in hexane before analysis. The resulting sterol-trimethylsilyl ethers were analysed using a Gas Chromatograph (GC) (Hewlett Packard/Agilent, Model 6890) coupled to a quadrupole mass spectrometer (Hewlett Packard 5973 mass selective detector). The GC was fitted with a CP-Sil 5 CB column (30m by 0.25mm; #CP8741 Agilent Technologies) set at 245°C and raised in 3.5°C/min steps to 265°C followed by 0.5°C/min steps to 310°C post injection. The injector with the transfer liner was set to 325°C, the interface to 300°C, and the ion source was maintained at 280°C. Helium with a flow rate of 1 mL/min was used as carrier gas. The full electron ionisation spectra were scanned in the range of 40 to 800 m/z. Published mass fragmentation patterns of sterol standards were used to confirm the identity of different sterols. The full protocol from derivatisation to analysis was also applied to the commercial standards to verify the accuracy of our technique. Our fragmentation patterns closely matched those in previous publications. The total peak area from the gas chromatogram was used to quantify sterol composition. Baseline analysis of the ingredients of the YM and Peptone media confirmed the absence of any sterols (Figure A in S1 File).

### Bioinformatic analysis and annotation of selected genes

The MetaCyc pathway pages (http://www.metacyc.org/) [37] were used to select sterol pathway genes from *Homo sapiens*, *Arabidopsis thaliana*, *Aphanomyces euteiches*, various fungi and other metazoans to search known enzymes against the *S*. *parasitica* and *P*. *infestans* genomes in the NCBI and Joint Genome Initiative (JGI) databases (Blast searches). Putative homologous genes were identified by having an E value greater than 1e^-5^, and compared with previously published *S*. *parasitica* sterol metabolic pathways for reference [18, 19]. For further confirmation of selected genes, the conserved domain(s) of each gene was investigated by NCBI Conserved Domain Search [38].

To predict activities for the enzymes encoded by the selected genes we first used the bioinformatics tool Blast2GO (B2G, https://www.blast2go.com/) [39]. B2G uses Blast [40] to identify sequences homologous to the input query sequence and assign a tentative Gene Ontology (GO) [41]. For the final analysis, selected gene features were additionally verified using NCBI Conserved Domain Search [38] and used for a new round of manual Blast searches to improve prediction of protein function. B2G and ExPASy-ENZYME (http://enzyme.expasy.org [42]) were used to identify the Enzyme Commission numbers, which were verified in the BRENDA enzyme database (http://www.brenda-enzymes.org [43]).

### RT-qPCR

Total RNA for qPCR analysis was isolated from mycelium using the RNeasy Plant Mini Kit (#74904 QIAGEN) and treated with RNase-free DNase for 20 min at 37°C (Ambion, TURBO DNA-free Kit). Extracted RNA was qualitatively visualised by agarose gel electrophoresis, and a NanoDrop 1000 spectrophotometer (Thermo Scientific) was used to quantify the total amount of RNA. Two μg template RNA was used for cDNA synthesis with the Maxima First Strand cDNA Synthesis Kit for RT-qPCR (#K1641 Thermo Fisher Scientific, Stockholm, Sweden).

QPCR primers for the genes under analysis were designed using Primer 3Plus (http://www.bioinformatics.nl/cgi-bin/primer3plus/primer3plus.cgi/) [44]. Primers were designed to achieve an amplification range between 100 and 120 bp, a GC content ranging between 50–60% and a length of 18 to 24 nucleotides. The primer melting temperatures were 60 ± 1°C and each primer pair was tested separately.

Gene expression levels were evaluated using the CFX96 Real-Time PCR detection system (BioRad). The reactions were performed using 5 μL of 2X iQ SYBR Green Supermix (Bio-Rad), 0.5 μM of each primer, 10ng cDNA, and nuclease-free water to a final volume of 10 μl in two technical replicates for each of three independent biological experiments.

RNA and no template control samples were also used as negative control for each primer pair. Cycle steps for qPCR analysis were 95°C for 3 min, followed by 44 cycles of 95°C for 10s denaturation, annealing at 60°C for 10s and extension at 72°C for 15s. The raw data were analysed using the CFX manager^™^ software (version 3.0; Bio-rad) which includes the algorithms to perform relative gene expression with normalization to multiple reference genes over multiple plates. Relative expression levels were calculated by normalizing the data to the geometric mean of three reference genes, which were selected from an expression stability analysis of eight reference genes (CFX manager^™^, version 3.0; Bio-rad). The reference genes used for *P*. *infestans* were ubiquitin-conjugating enzyme E2 (PITG_00505), 40S ribosomal protein (PITG_11766), and beta-tubulin (PITG_00156). The reference genes used for *S*. *parasitica* were ubiquitin-conjugating enzyme (UBC; SPRG_03371), glyceraldehyde-3-phosphate dehydrogenase (GADPH; SPRG_00090) and Elongation factor (Ef; SPRG_10439). The PCR efficiency for each gene was calculated using Real-time PCR Miner [45], and found to range from 85% to 99%.

### *Phytophthora infestans* sterol feeding study and growth assay

*P*. *infestans* strain T30-4 maintained on defined media supplemented with β-sitosterol and solidified with 2% plant agar was used for assessment of mycelial growth on sterol-supplemented media. Six- well plates (Sarstedt, Nümbrecht, NRW, Germany) were prepared with 2 mL of sterol free media as described above and supplemented with 50 mM of either β-sitosterol, lanosterol, cycloartenol, zymosterol, lathosterol, brassicasterol, desmosterol, stigmasterol, cholesterol, fucosterol, or ergosterol. An agar plug of 5 mm diameter covered by *P*. *infestans* mycelium was excised and used to inoculate each well after removing most of the agar without disrupting the mycelial surface. Plates were incubated at 25°C in the dark. Growth was followed by daily measurements of colony diameter in mm. Fourteen days after inoculation, the mycelia was harvested, washed 3 times with autoclaved and micro-filtered water, dried and snap frozen in liquid nitrogen prior to storage at -80°C. Sterols and RNA were then extracted as described above.

## Results

### Sterol biosynthesis in *Saprolegnia parasitica*

#### Sterol composition in *Saprolegnia parasitica*

**Fig 2:**
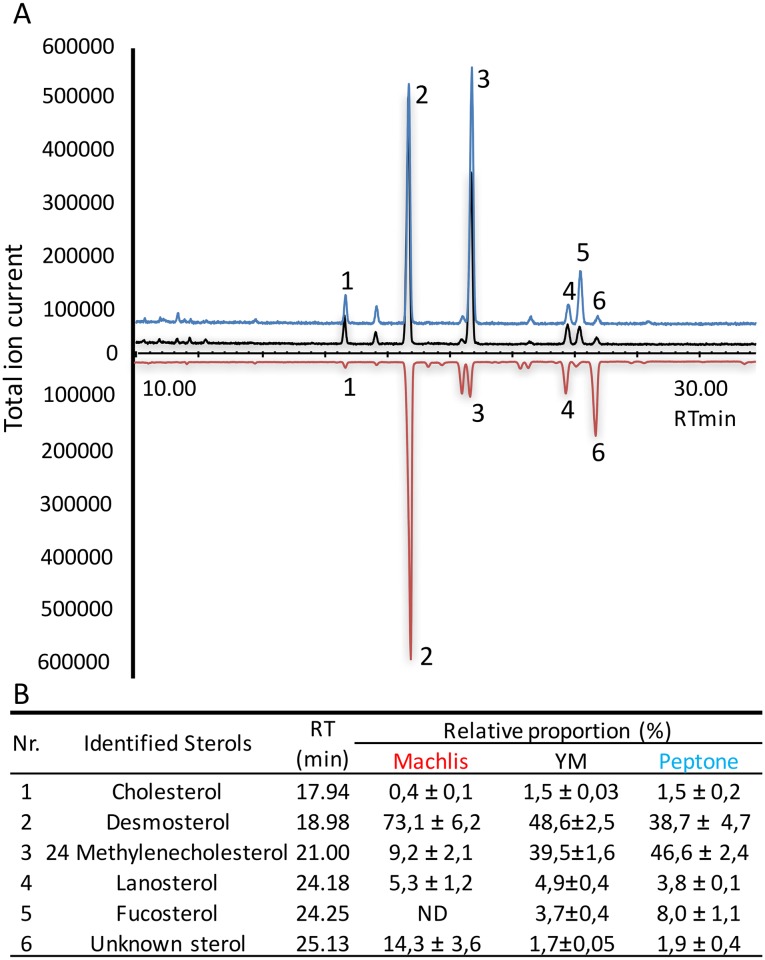
GC-MS analysis of *S*. *parasitica* sterol composition. Samples analysed were grown for 3 days in Machlis, YM, or Peptone media. GC-MS analyses were performed in triplicate. The dominant sterol was desmosterol for mycelia grown in Machlis (red trace in the GC chromatogram) and YM media (black trace), while 24-methylenecholesterol dominates in the mycelium grown in Peptone medium (blue trace). RT: Retention time.

*S*. *parasitica* mycelium was harvested after 3 days growth in defined Machlis medium, and an initial sterol profile analysis was performed by GC-MS. Lanosterol, desmosterol, cholesterol, 24-methylene cholesterol, and another unidentifiable sterol-like compound were detected (Fig 2). The sterol composition correlated quite well with the findings of Warrilow *et al*. [19], with the exception of fucosterol, which was not apparent in these initial investigations. The oomycete was subsequently grown in YM and Peptone media, and again harvested after 3 days growth. The sterol composition of these samples was markedly more similar to that previously reported for *S*. *parasitica* grown in YM medium [19], as fucosterol could be readily detected (Fig 2). The mycelial sterol profile was dominated by desmosterol in Machlis medium, and was also enriched in 24-methylene cholesterol in Peptone and YM media (Fig 2).

#### Bioinformatic mining for sterol related genes in *S*. *parasitica*

Sterol biosynthesis genes from *Homo sapiens*, plants and fungi were used to mine the *S*. *parasitica* genome for potential homologues. The functions of the identified genes were predicted using Blast2GO, and by manual nBLAST searching the sequence against the NCBI database for characterised genes (Table 1). Predictions were confirmed by analysing the conserved domains of protein sequences. In all, 24 genes could be identified which form complete MVA and sterol synthesis pathways (Fig 1), up to and including the fine modifications required to produce the end sterols (Fig 2). We also identified orthologous genes in *Aphanomyces invadans*, *Aphanomyces astaci*, and *Saprolegnia diclina* (Table 1), indicating a common sterol synthesis pathway in the Saprolegniales order.

**Table 1:** Gene sequences putatively involved in the MVA and sterol biosynthesis pathways of *S*. *parasitica*.

	Protein/Gene ID	Blast2GO sequence description	Closest orthologue prediction (GeneID)		Predicted function	EC number
			In Oomycetes	Excluding Stramenopiles		
MVA pathway	KDO35408.1/SPRG_00258	Acetyl-acetyltransferase	XP_008619526.1 (*S*. *diclina*)	WP_026946557 (*A*. *marincola*)	Acetyl-CoA C-acetyltransferase	2.3.1.9
	KDO31351.1/ SPRG_03968	Hydroxymethylglutaryl-synthase	XP_008875809.1 (*A*. *invadans*)	XP_004362382.1 (*D*. *fasciculatum*)	Hydroxymethylglutaryl-CoA synthase	2.3.3.10
	KDO33998.1/SPRG_01272	Hydroxymethylglutaryl-reductase	XP_008615517.1 (*S*. *diclina*)	XP_006660782.1 (*O*. *brachyantha*)	Hydroxymethylglutaryl-CoA reductase	1.1.1.34
	KDO29696.1/SPRG_05647	Mevalonate kinase	XP_008607725.1 (*S*. *diclina*)	EXX50833.1 (*R*. *irregularis*)	Mevalonate kinase	2.7.1.36
	KDO20867.1/SPRG_14098	Phosphomevalonate kinase	XP_008865054.1 (*A*. *invadans*)	CDH52938.1 (*L*. *corymbifera*)	Phosphomevalonate kinase	2.7.4.2
	KDO20414.1/SPRG_14352	IPP Δ-isomerase	XP_008869475.1 (*A*. *invadans*)	CAP17174.1 (*M*. *circinelloides*)	Isopentenyl-diphosphate Δ-isomerase	5.3.3.2
	KDO31253.1/SPRG_03870	Diphosphomevalonate decarboxylase	ETV89208 (*A*. *astaci*)	EIE86507.1 (*R*. *delemar*)	Diphosphomevalonate decarboxylase	4.1.1.33
	KDO29097.1/SPRG_06153	Solanesyl diphosphate synthase	XP_008870257.1 (*A*. *invadans*)	XP_005651593 (*C*. *subellipsoidea*)	Dimethylallyltranstransferase	2.5.1; 2.5.1.1
	KDO27624.1/SPRG_06894	Geranylgeranyl pyrophosphate synthase	XP_002900610 (*P*. *infestans*)	XP_004341343.1 (*A*. *castellanii*)	Geranylgeranyl diphosphate synthase	2.5.1.29
	KDO28185.1/SPRG_06233	Squalene synthase isoform 2	XP_008862737 (*A*. *invadans*)	XP_007429053 (*P*. *bivittatus*)	Squalene synthase	2.5.1.21
	KDO23327.1/SPRG_11641	Squalene monooxygenase-like	CAQ55983.1 (*A*. *euteiches*)	ADD17678.1 (*W*. *somnifera*)	Squalene monooxygenase	1.14.13.132
Sterol synthesis pathway	KDO22939.1/SPRG_17895	Cycloartenol synthase	CAQ55984.1 (*A*. *euteiches*)	XP_005702872 (*G*. *sulphuraria*)	Lanosterol synthase	5.4.99.8
	KDO22939.1/SPRG_11783	Cycloartenol synthase	CAQ55984.1 (*A*. *euteiches*)	XP_005702872 (*G*. *sulphuraria*)	Lanosterol synthase	5.4.99.8
	KDO18234.1/SPRG_16338	Oxidoreductase	SDRG_09361 (*S*. *diclina*)	XP_004345128 (*C*. *owczarzaki*)	Δ^3^ sterol keto reductase	-
	KDO30188.1/SPRG_04988	-	XP_008606306.1 (*S*. *diclina*)	ACT20729.1 (*D*. *pulex*)	Δ^24^ sterol reductase	1.3.1.72
	KDO25246.1/SPRG_09493	Obtusifoliol 14alpha-demethylase	CAQ55977.1 (*A*. *euteiches*)	XP_002981251 (*S*. *moellendorffii*)	Sterol 14α demethylase (CYP51)	1.14.13.70
	KDO35576.1/SPRG_00418	-	CAQ55985.1 (*A*. *euteiches*)	XP_005838841.1(*G*. *theta*)	Δ^14^ sterol reductase	1.3.1.70
	KDO33722.1/SPRG_01623	C4 methyl sterol oxidase	CAQ55986.1 (*A*. *euteiches*)	XP_003079549.1 (*O*. *tauri*)	Δ^4^ methyl sterol oxidase	1.14.13.72
	KDO34363.1/SPRG_01499	Sterol-4-α-carboxylate 3 (partial)	Hypothetical protein	XP_005095330.1 (*A*. *californica*)	Δ^3^ sterol dehydrogenase	1.1.1.170
	KDO20748.1/SPRG_13330	-	Hypothetical protein	XP_006674094.1 (*C*. *militaris*)	Δ^8^ sterol isomerase	5.3.3.5
	KDO22929.1/SPRG_11773	Lathosterol oxidase	XP_008619546.1 (*S*. *diclina*)	XP_004365724.1 (*C*. *owczarzaki*)	Δ^5^ sterol desaturase	1.14.21.6
	KDO15919.1/SPRG_18544	Lathosterol oxidase	XP_008619546.1 (*S*. *diclina*)	XP_004365724.1 (*C*. *owczarzaki*)	Δ^5^ sterol desaturase	1.14.21.6
	KDO35021.1/SPRG_01085	7-dehydrocholesterol reductase	XP_002900651.1 (*P*. *infestans*)	WP_013924762 (*P*. *acanthamoebae*)	Δ ^7^ sterol reductase	1.3.1.21
	KDO30241.1/SPRG_05001	-	CAQ55978.1 (*A*. *euteiches*)	XP_004336540 (*A*. *castellanii*)	Δ^24^ sterol methyltransferase	2.1.1.142

Closest orthologue predictions were made by comparing first only to other oomycetes, and then to all organisms except Stramenopiles. Predicted gene functions and the corresponding EC numbers are provided for each predicted activity, where available. All sterol biosynthetic gene orthologues from *Homo sapiens* and equivalent orthologues from plants and fungi in the MetaCyc metabolic pathway database were used for the BLASTp searches to predict enzyme function.

The *S*. *parasitica* genome includes two near-identical OSC genes involved in the cyclisation of 2,3-oxidosqualene (Fig 1) [32]. We recently confirmed by recombinant protein production and biochemical characterisation that the protein encoded by SPRG_11783 (*Sp*LASA) is a lanosterol synthase [32]. On this basis, we propose that sterol synthesis in *S*. *parasitica* proceeds via the formation of lanosterol, not cycloartenol. The product of the OSC reaction will pass to a sterol 14α demethylase (CYP51) encoded by SPRG_09493. Previous work by Warrilow *et al* showed that this enzyme likely acts on lanosterol [19], supporting our proposed lanosterol pathway.

For the subsequent enzymatic steps which convert 14-demethyllanosterol to a series of intermediate structures, we identified the same candidate genes as were presented in other recent papers describing lanosterol synthesis pathways [18, 19]. An activity not identified in previous reconstructions is the Δ^3^ sterol keto reductase required to produce some intermediate species. We tentatively predict this to be encoded by SPRG_16338, which has some homology to a human enzyme (29% sequence identity). These enzymes are poorly conserved between different classes of organisms, but domain analysis of SPRG_16338 does suggest oxidoreductase activity and NADP^+^ binding. For all subsequent enzymatic steps leading to the end sterols, we were able to identify candidate genes consistent with the findings of previous *S*. *parasitica* pathway reconstructions (Table 1) [18, 19].

#### Expression analysis of candidate *S*. *parasitica* sterol related genes

**Fig 3:**
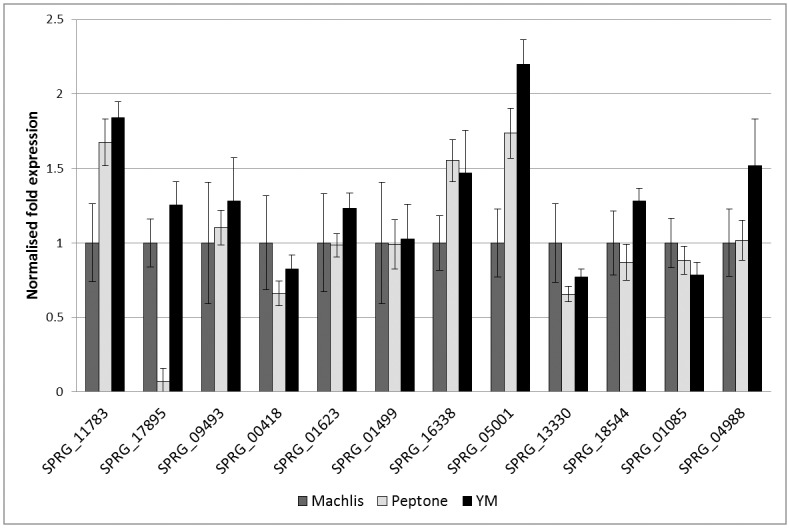
Gene expression analysis by qPCR of putative *S*.*parasitica* sterol related genes. Expression levels of each gene were standardised by comparison with the levels of expression of 3 housekeeping genes, and normalised to expression levels during growth on the defined Machlis medium. Genes are identified by their Gene ID. SPRG_11783: oxidosqualene cyclase (lanosterol synthase). SPRG_09493: CYP51 sterol 14α-demethylase. SPRG_00418: Δ^14^ sterol reductase. SPRG_01623: Δ^4^ methyl sterol oxidase. SPRG_05001: Δ ^24^ sterol methyltransferase. SPRG_13330: Δ^8^ sterol isomerase. SPRG_18544: Δ^5^ sterol desaturase. SPRG_01085: Δ^7^ sterol reductase. SPRG_04988: Δ^24^ sterol reductase. Three replicate samples were analysed in each case. Error bars represent one standard deviation from the mean.

qPCR analysis confirmed the expression of all identified genes during growth in all conditions. Expression levels in YM and Peptone media were normalised against levels in defined synthetic Machlis medium, and against a panel of housekeeping genes (Fig 3). Additionally, Figure B in S1 File shows gene expression levels of some MVA pathway genes, indicating that this pathway is utilised in *S*. *parasitica*. The level of expression of all genes tested varied depending upon the growth medium. We observed an increased transcriptional abundance of at least 1.5 fold for the lanosterol synthase gene SPRG_11783, the Δ^24^ sterol methyltransferase gene SPRG_05001, and the Δ^3^ sterol keto reductase gene SPRG_16338 during growth in YM and Peptone media, compared to Machlis samples (Fig 3). The Δ^24^ sterol reductase SPRG_04988 was upregulated more than 1.5 fold in YM samples compared to Peptone or Machlis samples (Fig 3). The greatest increase in expression compared to Machlis medium was seen for SPRG_05001 (1.75 fold in Peptone medium and 2.2 fold in YM medium).

### Assessing the potential for sterol conversion in *P*. *infestans*

#### *P*. *infestans* sterol feeding studies

Feeding studies confirmed the importance of exogenously provided sterols as a nutritional requirement for *P*. *infestans* [46]. Growth on sterol-free media was slow within the first days, and stopped completely within 7 to 10 days (Figure C in S1 File). Growth on various sterols was strong, while growth on the sterol precursor squalene was very poor, suggesting that this cannot compensate for sterols (Figure C in S1 File. GC-MS analysis of sterols extracted from *P*. *infestans* (Figure D in S1 File), showed an uptake of all sterols but no further conversion to other sterols. The β-sitosterol [47] utilised was contaminated with a small amount of campesterol (3.98%) (Figure D in S1 File). Within our range of accuracy, we identified the same ratio of β-sitosterol (95.95%) to campesterol (4.05%) in sterols extracted from mycelium. Our analysis of extracted sterols showed in some cases small additional peaks not present in the standards: these did not correspond to any known sterols or sterol-derivatives (Figure D in S1 File).

#### Bioinformatic mining for sterol related genes in *P*. *infestans*

*P*. *infestans* is known to be incapable of *de novo* sterol biosynthesis and to have a close homologue of the human Δ^7^ sterol reductase gene (PITG_13128) [12]. Building on this, we mined the *P*. *infestans* genome for additional genes related to the synthesis of sterols or sterol precursors. We identified genes responsible for the conserved regions of the MVA pathway up to the condensation of DMAPP and IPP into FPP (Fig 1), but could not identify candidate SQS or OSC genes (Table 2) [13, 20, 48]. We did uncover a putative Δ^5^ sterol desaturase (PITG_21426, Table 2). The products of these two genes might act in a cascade to modify sterols such as lathosterol and ergosterol (Figure E in S1 File), although neither of these sterols was converted by *P*. *infestans* (Figure D in S1 File).

**Table 2:** Gene sequences putatively involved in the mevalonate (MVA) pathway of *P*. *infestans*, or with roles in sterol modification.

	Protein/Gene ID	Blast2GO sequence description	Closest orthologue prediction (GeneID)		Predicted function	EC number
			In Oomycetes	Excluding Stramenopiles		
MVA pathway	XP_002908392.1/PITG_01783	Acetyl-acetyltransferase	XP_008619526 (S. diclina)	WP_010602725 (P. agri)	Acetyl-CoA C-acetyltransferase	2.3.1.9
	XP_002900378.1/PITG_12495	Hydroxymethylglutaryl-synthase	XP_008902107 (P. parasitica)	2F82_A (B. juncea)	Hydroxymethylglutaryl-CoA synthase	2.3.3.10
	XP_002901817.1/PITG_11028	Mevalonate kinase	ETM49801 (P. parasitica)	XP_007030863 (T. cacao)	Mevalonate kinase	2.7.1.36
	XP_002906345.1/PITG_03270	Phosphomevalonate kinase	XP_008911663 (P. parasitica)	CDH52938 (L. corymbifera)	Phosphomevalonate kinase	2.7.4.2
	XP_002898035.1/PITG_15778	Diphosphomevalonate decarboxylase	ETL30939 (P. parasitica)	XP_007468497 (L. vexillifer)	Diphosphomevalonate decarboxylase	4.1.1.33
	XP_002896895.1/PITG_16665	IPP Δ-isomerase	XP_008900922 (P. parasitica)	KDB11805 (V. virens)	Isopentenyl-diphosphate Δ-isomerase	5.3.3.2
	XP_002895983.1/PITG_20043	Solanesyl diphosphate synthase	XP_008870257 (A. invadans)	CDH51283 (L. corymbifera)	Solanesyl diphosphate synthase	2.5.1; 2.5.1.1
	XP_002900610.1/PITG_13077	Geranylgeranyl pyrophosphate synthetase	XP_008870257 (A.s invadans)	XP_004341343 (A. castellanii)	Dimethylallyltranstransferase	2.5.1.1
Sterol	XP_002894944.1/PITG_21426	C5 sterol desaturase	XP_008619546 (S. diclina)	NP_593135 (S. pombe)	Δ^5^ sterol desaturase	1.14.21.6
	XP_002900651.1/PITG_13128	7-dehydrocholesterol reductase	CAQ55987 (A. euteiches)	WP_013924762 (P. acanthamoebae)	Δ^7^ sterol reductase	1.3.1.21

Closest orthologue predictions were made by comparing first only to other oomycetes, and then to all organisms except Stramenopiles. Predicted gene functions and the corresponding EC numbers are provided for each predicted activity, where available. All sterol biosynthetic gene homologues from *Homo sapiens* and equivalent homologues from plants and fungi in the MetaCyc metabolic pathway database were used for the BLASTp searches to predict enzyme function.

#### Expression analysis of candidate *P*. *infestans* sterol related genes

**Fig 4:**
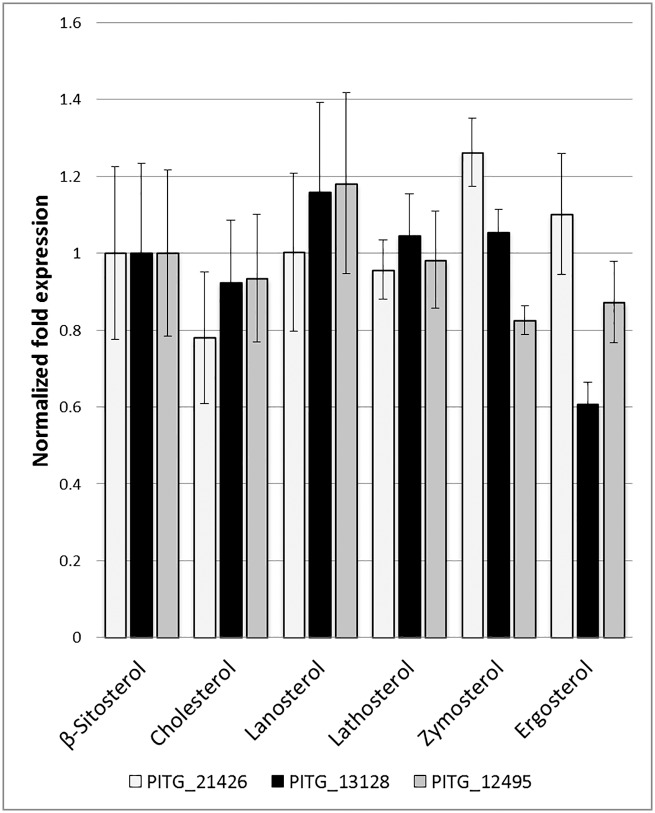
Gene expression analysis by qPCR of putative *P*. *infestans* sterol related genes. Expression levels of genes with possible roles in sterol modification were studied during growth in media supplemented with a range of different sterols. Transcript abundance was standardised by comparison with 3 housekeeping genes, and normalised to expression levels during growth on the host sterol β-sitosterol. Genes are identified by their Gene ID. PITG_21426: Δ^5^ sterol desaturase. PITG_13128: putative Δ^7^ sterol reductase. PITG_12495: HMG-CoA synthase. Three replicate experiments were performed in each case. Error bars represent one standard deviation from the mean.

Genes encoding a 3-hydroxy-3-methylglutaryl-coenzyme A (PITG_12495), a predicted Δ^5^ sterol desaturase (PITG_21426) and a predicted Δ^7^ sterol reductase (PITG_13128) were all expressed in *P*. *infestans* mycelium grown with β-sitosterol. Expression of these genes was also analysed for mycelium grown on lathosterol (a substrate for Δ^5^ sterol desaturase) and ergosterol (a substrate for Δ^7^ sterol reductase), as well as cholesterol, lanosterol and zymosterol (Fig 4). The data shown in Fig 4 strongly suggest an inability to modify sterols, since PITG_21426 and PITG_13128 showed no obvious increase in gene expression in the presence of their likely substrates. Indeed, PITG_13128 showed the lowest level of expression during growth on ergosterol.

## Discussion

### Reconstruction of a complete sterol synthesis pathway in *S*. *parasitica*

**Fig 5:**
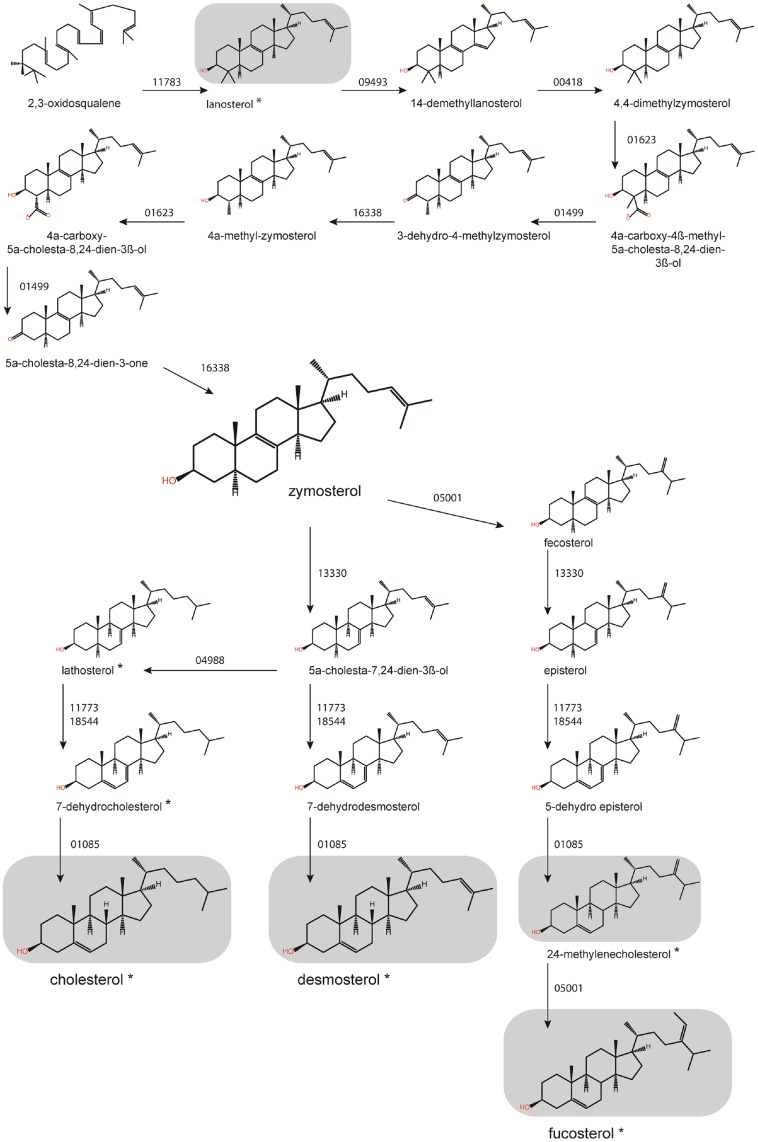
*In silico* reconstruction of the sterol synthesis pathway of *S*. *parasitica*. Synthesis steps are indicated by the gene (SPRG_xxxxx) predicted to encode the responsible enzyme. An asterisk (*) highlights those sterols which were previously extracted from Saprolegniales species. All sterols highlighted in grey boxes were identified in *S*. *parasitica* mycelium during this study. Zymosterol is shown larger than other sterols to highlight its role as a branching point between multiple synthetic routes.

Sterol synthesis pathways have recently been proposed for several Saprolegniales species [11, 12, 18, 19]. These reconstructions may be useful to identify targets in strategies to control the spread of these organisms. The pathway we propose for *S*. *parasitica* (Fig 5) is informed by a combined approach of bioinformatics to predict enzyme activity, GC-MS analysis of sterols extracted from mycelium, and qPCR analysis of gene expression levels. The pathway begins with the synthesis of a series of precursor molecules via the MVA pathway (Table 1; Figure B in S1 File) [7, 8, 49].

#### Committed sterol synthesis begins with the formation of lanosterol

Following the oxidation of squalene, 2,3-oxidosqualene is cyclised to create lanosterol. We recently demonstrated conclusively that the SPRG_11783 gene encodes a lanosterol synthase (*Sp*LASA, [32]), and this is further supported by the sterol profile of *S*. *parasitica* (Fig 2), where no cycloartenol was detected. Additional support derives from the predicted activity of the CYP51 sterol 14-alpha-demethylase (SPRG_09493): while this enzyme was shown to have similar binding affinities for lanosterol, eburicol and obtusifoliol, inhibition *in vivo* led to a high level of mycelial accumulation of lanosterol [19]. We therefore assume that SPRG_09493 encodes an enzyme acting on the lanosterol produced by *Sp*LASA [32].

In total, we predict genes associated with 11 enzymatic steps (Table 1, Fig 5, Table C in S1 File) converting lanosterol ultimately to cholesterol, desmosterol, 24-methylenecholesterol, and fucosterol. Each enzymatic step proposed by the MetaCyc pathway pages (http://www.metacyc.org/) (37), was separately investigated by a literature survey, and support was found for all cases (Table C in S1 File). After lanosterol synthesis, a CYP51 sterol 14α demethylase (SPRG_09493), Δ^14^ sterol reductase (SPRG_00418), Δ^4^ methyl sterol oxidase (SPRG_01623), Δ^3^ sterol dehydrogenase (SPRG_01499) and Δ^3^ sterol keto reductase (SPRG_16338) act in concert to produce zymosterol (Fig 5) before three alternative routes branch out. The Δ^3^ sterol keto reductase is newly identified in our pathway, and is responsible for a reduction at C3 to convert 3-dehydro-4-methyl-zymosterol into 4α-methyl-zymosterol.

#### Three synthetic pathways diverge after the formation of zymosterol

The major point of diversion between the three synthetic routes is the fate of zymosterol (Fig 5). First, a cascade of three enzymes acts sequentially to convert zymosterol to desmosterol: a Δ^8^ sterol isomerase (SPRG_13330) produces 5α-cholesta-7,24-dien-3β-ol, a Δ^5^ sterol desaturase (SPRG_17773 or SPRG_18554) converts this to 7-dehydrodesmosterol, and a Δ^7^ sterol reductase (SPRG_01085) produces desmosterol. Desmosterol was highly abundant in *S*. *parasitica* mycelium (Fig 2). If a Δ^24^ sterol methyltransferase (SPRG_05001) acts directly on zymosterol, producing fecosterol, the aforementioned three enzymes will produce episterol, 5-dehydro-episterol, and 24-methylenecholesterol (Fig 5). 24-Methylenecholesterol was also detected in the mycelium (Fig 2) and can act as a substrate for the Δ^24^ sterol methyltransferase, which converts it to fucosterol (found in mycelium from some media, Fig 2). Finally, if a Δ^24^ sterol reductase (SPRG_04988) acts on 5α-cholesta-7,24-dien-3β-ol, it will produce lathosterol: the synthetic pathway is diverted to produce 7-dehydrocholesterol, and finally cholesterol, which was detected to a small extent in the mycelium. The previous identification of lathosterol and 7-dehydrocholesterol, intermediates between zymosterol and cholesterol in *Aphanomyces euteiches* [12], strengthens the potential for such a route to cholesterol synthesis which does not require desmosterol as an intermediate.

Branching between these alternative routes is mediated by SPRG_05001 and SPRG_04988 (Fig 5), which encode enzymes to transfer or reduce the side chain at position C24. While in higher plants and diatoms, the Δ^24^ sterol methyltransferase appears to act on cycloartenol [1, 20, 50, 51], the *Saccharomyces cerevisiae* enzyme converts zymosterol to fecosterol [51–53], and we predict the same activity in *S*. *parasitica* (Fig 5). Indeed, without the action of the Δ^24^ sterol methyltransferase (SPRG_05001), cholesterol and desmosterol would be the only end products of the biosynthetic pathway, while we have clearly shown that *S*. *parasitica* mycelium can also contain large proportions of fucosterol and its precursor 24-methylenecholesterol.

#### Cross-talk between the three primary routes of synthesis

**Fig 6:**
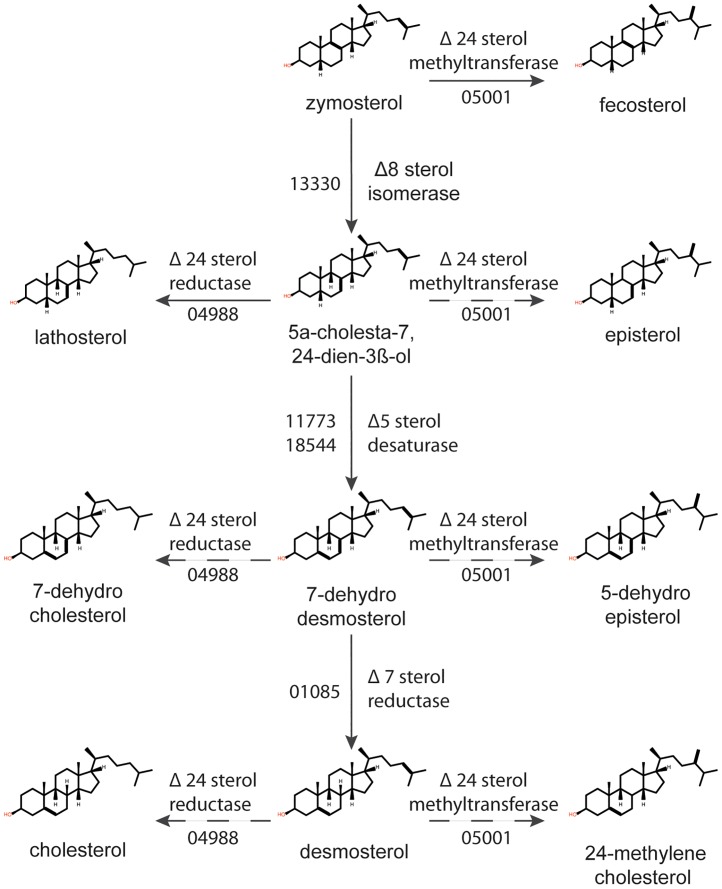
Potential cross-talk between the three major pathways of sterol biosynthesis in *Saprolegnia parasitica*. The flow-chart shows how the enzymes encoded by SPRG_05001 (Δ^24^ sterol methyltransferase) and SPRG_04988 (Δ^24^ sterol reductase) may be capable of acting on multiple different sterols, creating cross-talk between the three synthetic routes described in Fig 5. Solid arrows show the main synthesis routes depicted in Fig 5, while the dashed arrows indicate predicted points of cross-talk between these routes.

Although zymosterol is the preferred substrate for fungal Δ^24^ sterol methyltransferases, these enzymes can also act on 5α-cholesta-7,24-dien-3β-ol, 7-dehydrodesmosterol, desmosterol and others [6, 51, 54]. We hypothesise that the action of a catalytically flexible Δ^24^ sterol methyltransferase in *S*. *parasitica* will allow for multiple potential routes to fucosterol production, as intermediates from the pathways leading to cholesterol and desmosterol may be diverted towards fucosterol synthesis, as illustrated in Fig 6. Desmosterol is a known possible intermediate in cholesterol biosynthesis [55], and while the Δ^24^ sterol reductase (SPRG_04988) could convert desmosterol into cholesterol, we have shown that cholesterol is not very abundant in *S*. *parasitica* mycelium, despite expression of the SPRG_04988 gene. While the intermediates lathosterol and 7-dehydrocholesterol could not be detected in *S*. *parasitica*, there is some support for this multiple functionality of the Δ^24^ sterol reductase in the identification of these sterols in *A*. *euteiches* [12].

As the Δ^24^ sterol methyltransferase is predicted to act at multiple stages to divert synthesis towards fucosterol, one would expect that high expression of SPRG_05001 would lead to a significant shift in the profile of end sterols. Indeed, our data on mycelium grown in different media show that the highest levels of SPRG_05001 expression correlate with the highest proportions of fucosterol and its immediate precursor 24-methylenecholesterol. However, fucosterol was absent in Machlis medium-grown mycelium, despite expression of the gene. It may be that the enzyme must be specifically targeted to a cellular area to perform this function, and the weak growth in Machlis medium impairs this process.

#### *S*. *parasitica* adapts its sterol profile in response to environmental changes

*S*. *parasitica* mycelium grown in different media shows high variability in sterol composition (Fig 2). In defined Machlis medium, the synthetic route leading to desmosterol production dominates. Conversely, in YM and Peptone media, the fucosterol route dominates, although it tends to terminate with the production of 24-methylenecholesterol. Our gene expression analyses show complementary transcriptional changes based on growth media conditions (Fig 3). By visual observation, growth of *S*. *parasitica* on Machlis medium produces a denser mycelium mat, and a reduced amount of biomass compared to the other media. This may be due to slightly nutrient-poor conditions in Machlis medium. While YM and peptone media provide complex sugars, peptides, and amino acids, the synthetic medium contains only glucose, methionine, glutamic acid and thiamine. The resulting increase in metabolic difficulty might be a factor influencing the sterol synthesis pathway taken. In the synthetic medium, it is feasible that *S*. *parasitica* has different membrane requirements or reproductive behaviours, which will require a different sterol profile. To our knowledge, adaptation of sterol synthesis to different growth media has not been demonstrated earlier for *S*. *parasitica*. This implies that the local environmental conditions and host tissue during infection may impact the sterol profile of the invading oomycete.

### Sterol requirements of *P*. *infestans*

Sterol feeding studies of Peronosporales species have previously demonstrated the conversion of small proportions of provided lanosterol or cycloartenol into 1–5% cholesterol [27, 28, 56]. Conversion of these sterols to cholesterol would likely require at least 9 enzymes performing multiple conversion steps. Our own bioinformatic mining of the *P*. *infestans* genome revealed only two such genes, which putatively encode a Δ^5^ sterol desaturase (PITG_21426) which may act on lathosterol, and a Δ^7^ sterol reductase (PITG_13128) which may act on ergosterol (Figure E in S1 File). We initiated a sterol feeding study of *P*. *infestans* to explicitly probe the likely functions of these enzymes, and to assess the capacity of the organism to take up and modify specific sterols which are likely substrates of these enzymes. As in previous studies [14, 16, 17, 46], sterol provision was an absolute requirement for growth. Additionally, while all of the sterols tested were taken up and supported growth, we found no evidence of conversion to different sterols in any case. This includes lathosterol and ergosterol, which were selected to specifically probe for activity of the putative enzymes. Although we confirmed expression of both PITG_21426 and PITG_13128, neither was expressed at a much higher level in the presence of a putative substrate (Fig 4). Our data therefore indicate that *in vivo* sterol modification does not occur in *P*. *infestans* to a detectable extent, even though the required genes are expressed (Fig 4). The true functions of the genes encoding a Δ^5^ sterol desaturase and a Δ^7^ sterol reductase still need to be elucidated, but it is conceivable that they might have roles in host fitness or sterol parasitism [57].

### Sterol modification as a survival adaptation and drug target

The two model species of pathogenic oomycetes we have studied take very different approaches to sterol acquisition. Both are highly successful pathogens in their natural environments, as evidenced by the significant impacts they make on agriculture and aquaculture. Yet it is intriguing to consider whether sterol autotrophy or heterotrophy offer different survival advantages.

A potential advantage to survival for the oomycetes is an ability to evade host defence mechanisms. In plants, sterols generally play an important role in host resistance to pathogen infection [58, 59]. It is tempting to suggest that *P*. *infestans*, which produces no ‘alien’ sterols detectable by the plant, is adapted to survive in its host. However, oomycete sterol uptake from the host, necessary for *P*. *infestans* survival [60], is typically performed by carrier proteins known as elicitins [61, 62]. It is unknown precisely how oomycete elicitins affect plant behaviour, but they are named specifically for their ability to elicit a high level of defensive response in plants [63–65]. These are abundant in the mycelium of Peronosporales species [62, 66], and likely compensate for an inability to synthesise sterols [62]. The elicitins could therefore represent a promising drug target for disruption of nutrient acquisition in the oomycete.

Preliminary efforts to control oomycete growth via the direct inhibition of sterol synthesis show some early promise. The Δ^24^ sterol methyltransferase encoded by SPRG_05001 may be a suitable target for inhibition in *S*. *parasitica*, as it is not found in animals [6]. However, as this gene is expressed at very different levels in different media, its expression level should be studied in host tissues. The multiple pathways of sterol synthesis we propose might even allow for effective compensation for the loss of this activity. Indeed, the adaptive nature of sterol synthesis in *S*. *parasitica* may be a significant driver of the organism’s success in its environment. Fungicide-mediated inhibition of the CYP51 enzyme, which acts before the zymosterol branching point, has already shown some success as a control treatment [19]. To complicate matters, the presence of an elicitin-encoding gene in *Aphanomyces euteiches* suggests that the inhibition of sterol synthesis could be compensated for in some Saprolegniales by increased sterol uptake from the host [11, 12].

## Conclusions and outlook

Since sterols are vital for oomycete survival, we emphasise the importance of these molecules as potential drug targets in control strategies. However, the acquisition of these lipids differs greatly between the Saprolegniales and Peronosporales, which will limit the range of applicability of any sterol-targeting treatments. We have shown that the fish pathogen *S*. *parasitica* can produce a range of different sterols. The sterol profile in the mycelium is altered by changing the growth medium, which correlates with alterations in the level of expression of sterol synthesising genes. Our data indicate that the profile of sterols synthesised in a natural setting is likely to be host-dependent, and that *S*. *parasitica* could compensate for the loss of one sterol synthesis route. This observation should inform the design of control strategies. Nonetheless, the complex sterol synthesis pathway we present for *S*. *parasitica* may lead to the identification of some specific targets for inhibition. We have additionally confirmed that in our experimental set-up *P*. *infestans* mycelial growth is absolutely dependent upon sterol uptake, and found no evidence that the organism can modify exogenous sterols despite possessing some genes for sterol modification. We expect that future investigations into the inhibition of sterol uptake by *P*. *infestans* might be more successful by focussing on the elicitin proteins, which are likely key to sterol uptake by this devastating pathogen.

## Supporting information

S1 File **Table A Primers used in qPCR analysis of gene expression in *Saprolegnia parasitica*. Table B Primers used in qPCR analysis of gene expression in *Phytophthora infestans*. Figure A GC-MS analysis of media and media components utilised for cultivation of *Saprolegnia parasitica*.** Sterols were shown to be absent in all media and media components utilised in the study. A: GC chromatogram of peptone. B: GC chromatogram of Peptone medium. C: GC chromatogram of yeast mold. D: GC chromatogram of YM medium. **Figure B Gene expression analysis by qPCR of *S*.*parasitica* genes with predicted roles in the MVA pathway.** Expression levels of each gene were standardised against that of a panel of housekeeping genes, and normalised to expression during growth on the defined Machlis medium. Different growth media are indicated in different colours (blue: Machlis medium, red: Peptone medium, yellow: Yeast-Mold medium). Abbreviations used in the predicted enzyme names are as follows: SP (*Saprolegnia parasitica*), C14SR (Δ14 sterol reductase), HMG (hydroxymethylglutaryl-CoA synthase), HMGCAR (hydroxymethylglutaryl-CoA reductase), IDI (isopentenyl-diphosphate isomerase), MVD (mevalonate disphosphate decarboxylase/ diphosphomevalonate decarboxylase), MVK (mevalonate kinase), PMK (phosphomevalonate kinase), SQE (squalene monooxigenase) and SQS (squalene synthase). Three replicate experiments were performed in each case. **Figure C Growth of *Phytophthora infestans* on synthetic media supplemented with different sterols.** The colour key indicates the sterol or sterol precursor present in each experiment. The control culture contained no sterols and led to severely reduced growth. Growth on the sterol precursor squalene was also very poor. A 5 mm plug of excised mycelia was used to inoculate each growth medium. Six replicate experiments were performed in each case. **Figure D Various sterols fed to *P*. *infestans*, and subsequently extracted from mycelia, as investigated by Gas Chromatography coupled to Mass Spectrometry (GC-MS).** Each box represents a different sterol used in the feeding study, where the black spectra indicate sterol standards used for feeding and the red spectra indicate sterols extracted from 14-day-old *P*. *infestans* mycelia. The major peaks in the GC spectra are indicated in each case by an asterix (*), and the accompanying MS spectra show the important identifying m/z values in the fragmentation pattern. For β-sitosterol (top), two peaks can be seen in the gas chromatogram, a minor peak corresponding to campesterol, and a major peak corresponding to β-sitosterol (both confirmed by MS fragmentation analysis). Other small peaks could be observed, but fragmentation patterns showed that these did not correlate with any sterols, and are likely extract residues from the SPE column preparations. Figure E Putative sterol modifying activities of a Δ^5^ sterol desaturase and a Δ^7^ sterol reductase. **A: predicted conversion of lathosterol to 7-dehydrocholesterol by a Δ**^**5**^ **sterol desaturase. B: predicted conversion of ergosterol to brassicasterol by a Δ**^**7**^ **sterol reductase.**
(DOCX)

## Abbreviations

PMVK: phosphomevalonate kinase
IDI: isopentenyl diphosphate isomerase
SQS: squalene synthase
SqE: squalene epoxidase
OSC: oxidosqualene cyclase
MVA: mevalonate pathway
MEP: plastidic methylerythritol phosphate pathway
ACCOA: acetyl-CoA
AACCOA: aceto-acetyl-CoA
HMGCOA: 3-hydroxy-3-methylglutaryl-coenzyme A
MVA: mevalonate
MVAP: mevalonate phosphate
MVAPP: mevalonate diphosphate
IPP: isopentenyl diphosphate
DMAPP: dimethylallyl pyrophosphate
GPP: geranyl diphosphate
FPP: farnesyl diphosphate
PSDP: presqualene diphosphate
