# Supplementary material for: Comparative analysis of sterol acquisition in the oomycetes Saprolegnia parasitica and Phytophthora infestans
Source: PLoS One. 2017 Feb 2;12(2):e0170873. doi: 10.1371/journal.pone.0170873 (PMC5289490; doi:10.1371/journal.pone.0170873)
Supplement: S1 File — Table A Primers used in qPCR analysis of gene expression in Saprolegnia parasitica. Table B Primers used in qPCR analysis of gene expression in Phytophthora infestans. Figure A GC-MS analysis of media and media components utilised for cultivation of Saprolegnia parasitica. Sterols were shown to be absent in all media and media components utilised in the study. A: GC chromatogram of peptone. B: GC chromatogram of Peptone medium. C: GC chromatogram of yeast mold. D: GC chromatogram of YM medium. Figure B Gene expression analysis by qPCR of S.parasitica genes with predicted roles in the MVA pathway. Expression levels of each gene were standardised against that of a panel of housekeeping genes, and normalised to expression during growth on the defined Machlis medium. Different growth media are indicated in different colours (blue: Machlis medium, red: Peptone medium, yellow: Yeast-Mold medium). Abbreviations used in the predicted enzyme names are as follows: SP (Saprolegnia parasitica), C14SR (Δ14 sterol reductase), HMG (hydroxymethylglutaryl-CoA synthase), HMGCAR (hydroxymethylglutaryl-CoA reductase), IDI (isopentenyl-diphosphate isomerase), MVD (mevalonate disphosphate decarboxylase/ diphosphomevalonate decarboxylase), MVK (mevalonate kinase), PMK (phosphomevalonate kinase), SQE (squalene monooxigenase) and SQS (squalene synthase). Three replicate experiments were performed in each case. Figure C Growth of Phytophthora infestans on synthetic media supplemented with different sterols. The colour key indicates the sterol or sterol precursor present in each experiment. The control culture contained no sterols and led to severely reduced growth. Growth on the sterol precursor squalene was also very poor. A 5 mm plug of excised mycelia was used to inoculate each growth medium. Six replicate experiments were performed in each case. Figure D Various sterols fed to P. infestans, and subsequently extracted from mycelia, as investigated by Gas Chromatography coupled to Mass Spe [file pone.0170873.s001.docx]

# S1 Supplementary Information

Table A Primers used in qPCR analysis of gene expression in *Saprolegnia parasitica.*

| ***Saprolegnia parasitica*** | | | | |
| --- | --- | --- | --- | --- |
| Gene ID | Predicted function | Forward primer | Reverse primer | Product size (bp) |
| SPRG_03968 | Hydroxymethylglutaryl-CoA synthase | CAGCAGTCCAAGACCAAGAA | GTAGTTGGCCGGGAAGTAGA | 108 |
| SPRG_01272 | Hydroxymethylglutaryl-CoA reductase | GTGCAAGGCTACGACTATGC | TTTCGCCGTTGACTTGGATG | 109 |
| SPRG_03870 | Diphosphomevalonate decarboxylase | AGCAGCTCAAGACGGTCAC | CCTGGATACGCTTGTTGTTG | 105 |
| SPRG_05647 | Mevalonate kinase | CCACTTCAACGACTGACGAC | CCATAGACGACTGCATGCTC | 113 |
| SPRG_14098 | Phosphomevalonate kinase | CCAAGAACATGCAGATCGTC | GTTTAGGTGCACCCACGAG | 107 |
| SPRG_14352 | Isopentenyl-diphosphate Δ-isomerase | GGAGGAGATGTGCATCCAG | GAAGAGGAAGACGGAGAACG | 115 |
| SPRG_06233 | Squalene synthase | CGGACACTACATGGTGCTTC | CAGGTAAAACACGCAGATCG | 110 |
| SPRG_11641 | Squalene monooxygenase | GTGTACCTCCTCGGCTTTGT | CCGCCAATGATAATGACATC | 110 |
| SPRG_11783 | Lanosterol synthase | GACGATCTTCTTGCGACCAC | CCCGCGACTCTTTTCATACG | 114 |
| SPRG_09493 | Sterol 14α demethylase (CYP51) | CCAGGACTTGACGTTCCTCA | GGGCGTCATGAGCTGGTATA | 103 |
| SPRG_00418 | Δ^14^ sterol reductase | CTCACGTACAAGGTCAACGG | GCTGGAGGTAGTGGTCGTAG | 112 |
| SPRG_01623 | Δ^4^ methyl sterol oxidase | GCTGGACAACTACTCGGAGT | GAATGTAGTCGAGCGCCAAG | 107 |
| SPRG_01499 | Δ^3^ sterol dehydrogenase | TCCTCTTTGTAAGCTCGGCC | GGAATTTGGTCCGGCAGTAG | 110 |
| SPRG_16338 | Δ^3^ sterol keto reductase | GTTCAGCGCCAAGTTTGAG | GGTTGACACCAAACTGCATC | 110 |
| SPRG_05001 | Δ^24^ sterol methyltransferase | ACGCTCGAGTTCCTCAAGAT | GACGGCGTAAAGATGTCCA | 113 |
| SPRG_13330 | Δ^8^ sterol isomerase | GCGCACTTTACGATGCTCAT | CTCGAGGTAGCTGTGGATGT | 105 |
| SPRG_18544 | Δ^5^ sterol desaturase | CGCTCCAGAAAGTCGTGTAC | GATCTTCTCAAACCAGCGCG | 113 |
| SPRG_01085 | Δ ^7^ sterol reductase | AGAAGCCCGAGTACATCACC | TCGGGGATGTAGTGGAAGTG | 115 |
| SPRG_04988 | Δ^24^ sterol reductase | GTTCTTCAAGCACGTCGAGG | AAGCTCCCAAAAGATGCTGC | 109 |
| SPRG_03371 | Ubiquitin-conjugating enzyme (UBC)* | CAAGATGTTCCACCCCAAC | GACTGGATCGAGGTCAGGAT | 111 |
| SPRG_00090 | glyceraldehyde-3-phosphate dehydrogenase (GAPDH)* | TCGTGGTACGACAACGAGTGG | TGATGCTGTCGCGTCTTCTTGC | 101 |
| SPRG_10439 | Elongation factor (Ef)* | CATGATCACGGTTCGAGGC | CGAGACTTCCTCCTTGATTTCCT | 127 |

*Reference genes used to normalise expression levels between replicate samples.

Table B Primers used in qPCR analysis of gene expression in *Phytophthora infestans.*

| ***Phytophthora infestans*** | | | | |
| --- | --- | --- | --- | --- |
| Gene ID | Predicted function | Forward primer | Reverse primer | Product size (bp) |
| PITG_12495 | Hydroxymethylglutaryl-CoA synthase | CGCGACTCTACTGGTGTCAT | TACGTAGACTCGCCCTCCTT | 107 |
| PITG_21426 | Δ^5^ sterol desaturase | TGCTGATCTACTGGCTCCAC | CGTGAGATGCAAACGGAGAG | 105 |
| PITG_13128 | Δ^7^ sterol reductase | GTGGCCAAGTTCTTCTGGTG | GACGGCACCCATACTAGACA | 107 |
| PITG_00505 | Ubiquitin-conjugating enzyme E2 (Ubc)* | CTTCACGACCCGCATTTACC | AGCAGCACCTTCGAGATAGT | 108 |
| PITG_11766 | 40S ribosomal protein (40S)* | GCTGACCTGAACAAGGACGA | TCCATGCCGTGAAATCCAGT | 101 |
| PITG_00156 | Beta-tubulin* | TGAACTCGGACCTGCGTAAG | GCGATGTCAGAGGAGCGAAA | 90 |

*Reference genes used to normalise expression levels between replicate samples.

Table C Supporting information for each step of the proposed sterol synthesis pathway for *S. parasitica*, shown in Figure 5. Each sterol conversion is proposed by the BioCyc database (37) and additionally supported by a literature survey.

| **Protein/Gene ID** | **Predicted function** | **EC number** | **Substrate** | **Product** | **Functional characterized** | **Predicted conversion based on similar substrate, or prediction after computer modeling** |
| --- | --- | --- | --- | --- | --- | --- |
| KDO22939.1  SPRG_17895;  KDO22939.1  SPRG_11783 | Lanosterol synthase | 5.4.99.8 | 2,3-oxidosqualene | Lanosterol | Dahlin et al. (2016) Front Microbiol 7:1802. |  |
| KDO18234.1  SPRG_16338 | Δ3 sterol keto reductase | 1.1.1.270 | 3-dehydro-4-methylzymosterol | 4α-methyl-zymosterol |  | Gachotte et al. (1999). Proc Natl Acad Sci USA 96:12655-60. |
|  |  |  | 5α-cholesta-8,24-dien-3-one | Zymosterol |  |  |
| KDO30188.1  SPRG_04988 | Δ24 sterol reductase | 1.3.1.72 | 5alpha-cholest-7-en-3beta-ol | lathosterol |  | Soo-Han & Young-Ki.(1997) Biochemical Journal 326:609-16. |
|  |  |  | 7-dehydrodesmosterol | 7-dehydrocholesterol | Soo-Han & Young-Ki.(1997) Biochem J 326:609-16. | Soo-Han & Young-Ki.(1997) Biochem J 326:609-16. |
|  |  |  | desmosterol | cholesterol | Pedretti et al. (2008) Steroids 73:708-19 |  |
| KDO25246.1  SPRG_09493 | Sterol 14α demethylase | 1.14.13.70 | lanosterol | 14-dimethyllanosterol | Strömstedt et al. (1996) Arch Biochem Biophys 329:73-81. |  |
| KDO35576.1  SPRG_00418 | Δ14 sterol reductase | 1.3.1.70 | 14-demethyllanosterol | 4,4-dimethylzymosterol | Paik et al. (1984) J Biol Chem 259:13413-23. |  |
| KDO33722.1  SPRG_01623 | Δ4 methyl sterol oxidase | 1.14.13.72 | 4α-methyl-zymosterol | 4α-hydroxymethyl-5α- cholesta-8,24-dien-3β-ol |  | Latendresse M. (2013)* |
| KDO34363.1  SPRG_01499 | Δ3 sterol dehydrogenase | 1.1.1.170 | 4α-carboxy-4β-methyl- 5α-cholesta-8,24-dien-3β-ol | 5α-cholesta-8,24-dien-3-one |  | Gachotte et al. (1998). Proc Natl Acad Sci USA 95:13794-9. |
| KDO20748.1  SPRG_13330 | Δ8 sterol isomerase | 5.3.3.5 | zymosterol | 5α-cholesta-7,24-dien-3β-ol |  | Caspi et al. (2014) Nucleic Acids Res 42:459-71;  Arthington,et al. (1991). Gene 102:39-44. |
|  |  |  | fecosterol | episterol |  |  |
| KDO22929.1  SPRG_11773; KDO15919.1  SPRG_18544 | Δ5 sterol desaturase | 1.14.21.6 | 5α-cholesta-7,24-dien-3β-ol | 7-dehydrocholesterol |  | Taton & Rahier (1996). Arch Biochem Biophys 325:279-88; Taton et al. (2000) Biochem 39:701–11 |
|  |  |  | 5α-cholesta-7,24-dien-3β-ol | 7-dehydrodesmosterol |  |  |
|  |  |  | episterol | 5-dehydro episterol |  |  |
| KDO35021.1  SPRG_01085 | Δ 7 sterol reductase | 1.3.1.21 | 7-dehydrocholesterol | Cholesterol | Moebius et al. (1998). Proc Natl Acad Sci USA 95:1899-902. | Taton & Rahier (1996). Arch Biochem Biophys 325:279-88;  Dempsey et al (1964). J Biol Chem 239:1381-7. |
|  |  |  | 7-dehydrodesmosterol | Desmosterol |  |  |
|  |  |  | episterol | 5-dehydro episterol |  |  |
| KDO30241.1  SPRG_05001 | Δ24 sterol methyl- transferase | 2.1.1.41 | Zymosterol | Fecosterol | Nes et al. (1998) Arch Biochem Biophys 353:297-311. | Nes et al. (1999) J Org Chem 64:1535-42. |
|  |  |  | 5α-cholesta-7,24-dien-3β-ol | episterol |  |  |
|  |  |  | 7-dehydrodesmosterol | 5-dehydro episterol |  |  |
|  |  |  | 24-methylenecholesterol | fecosterol |  |  |


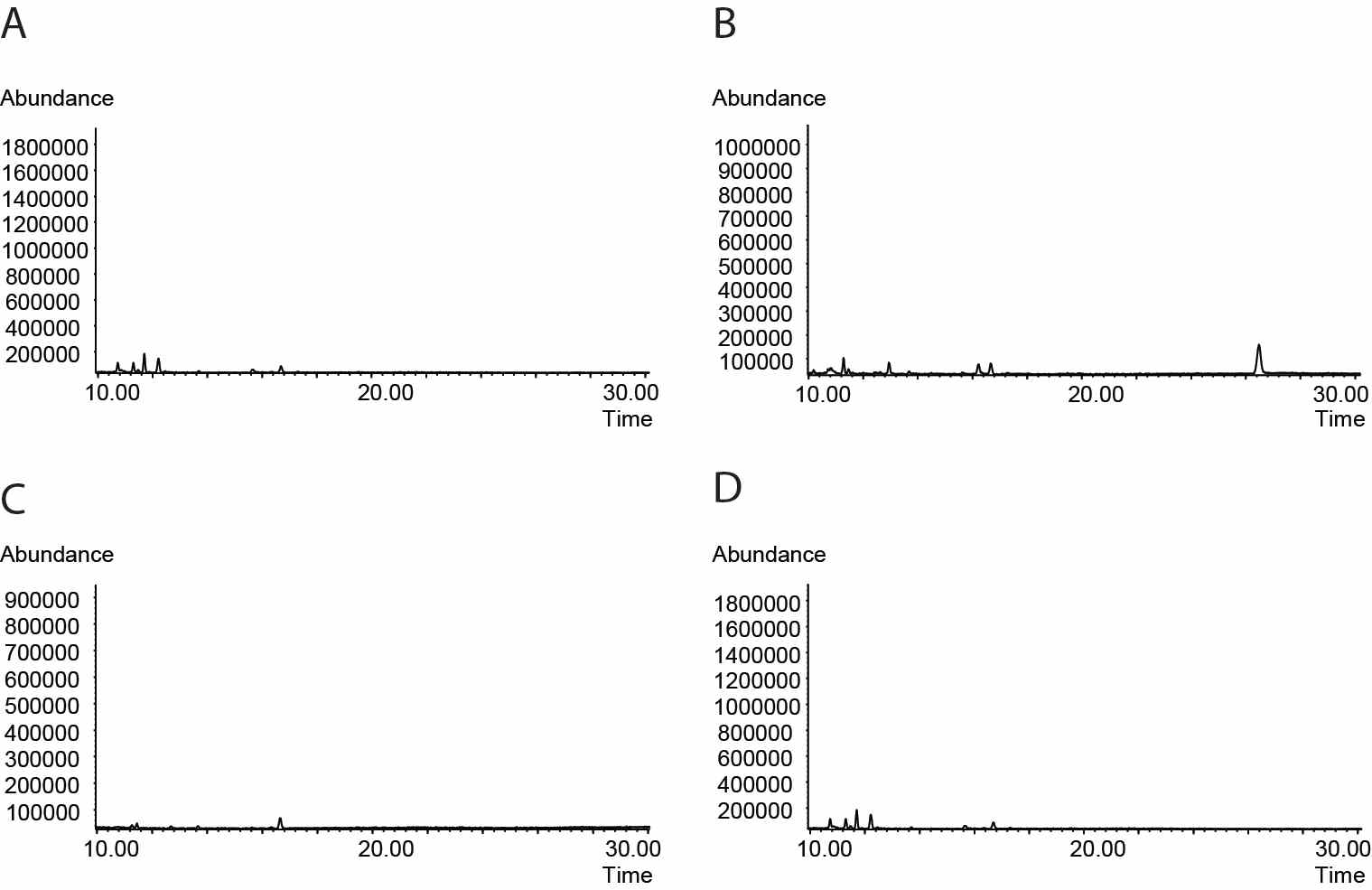


Figure A GC-MS analysis of media and media components utilised for cultivation of *Saprolegnia parasitica.*

Sterols were shown to be absent in all media and media components utilised in the study. A: GC chromatogram of peptone. B: GC chromatogram of Peptone medium. C: GC chromatogram of yeast mold. D: GC chromatogram of YM medium.


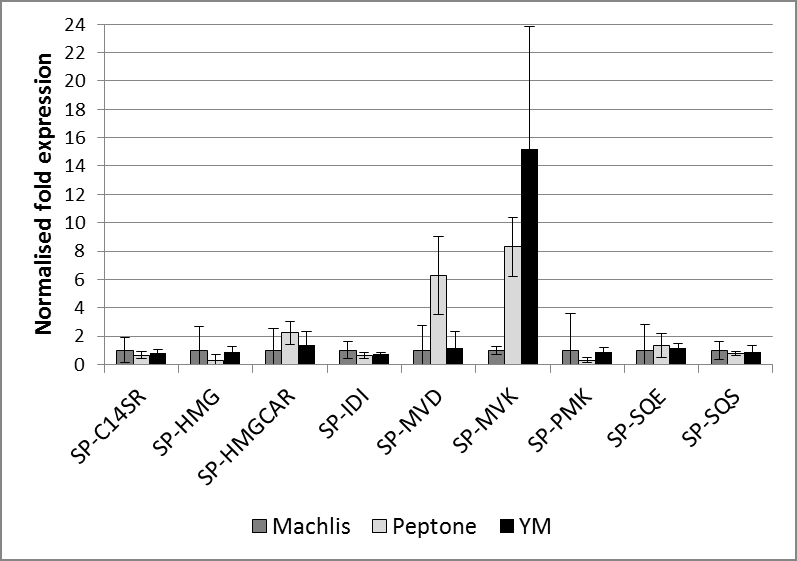


Figure B Gene expression analysis by qPCR of *S.parasitica* genes with predicted roles in the MVA pathway.

Expression levels of each gene were standardised against that of a panel of housekeeping genes, and normalised to expression during growth on the defined Machlis medium. Different growth media are indicated in different colours (blue: Machlis medium, red: Peptone medium, yellow: Yeast-Mold medium). Abbreviations used in the predicted enzyme names are as follows: SP (*Saprolegnia parasitica*), C14SR (Δ14 sterol reductase), HMG (hydroxymethylglutaryl-CoA synthase), HMGCAR (hydroxymethylglutaryl-CoA reductase), IDI (isopentenyl-diphosphate isomerase), MVD (mevalonate disphosphate decarboxylase/ diphosphomevalonate decarboxylase), MVK (mevalonate kinase), PMK (phosphomevalonate kinase), SQE (squalene monooxigenase) and SQS (squalene synthase). Three replicate experiments were performed in each case. Error bars represent one standard deviation from the mean.


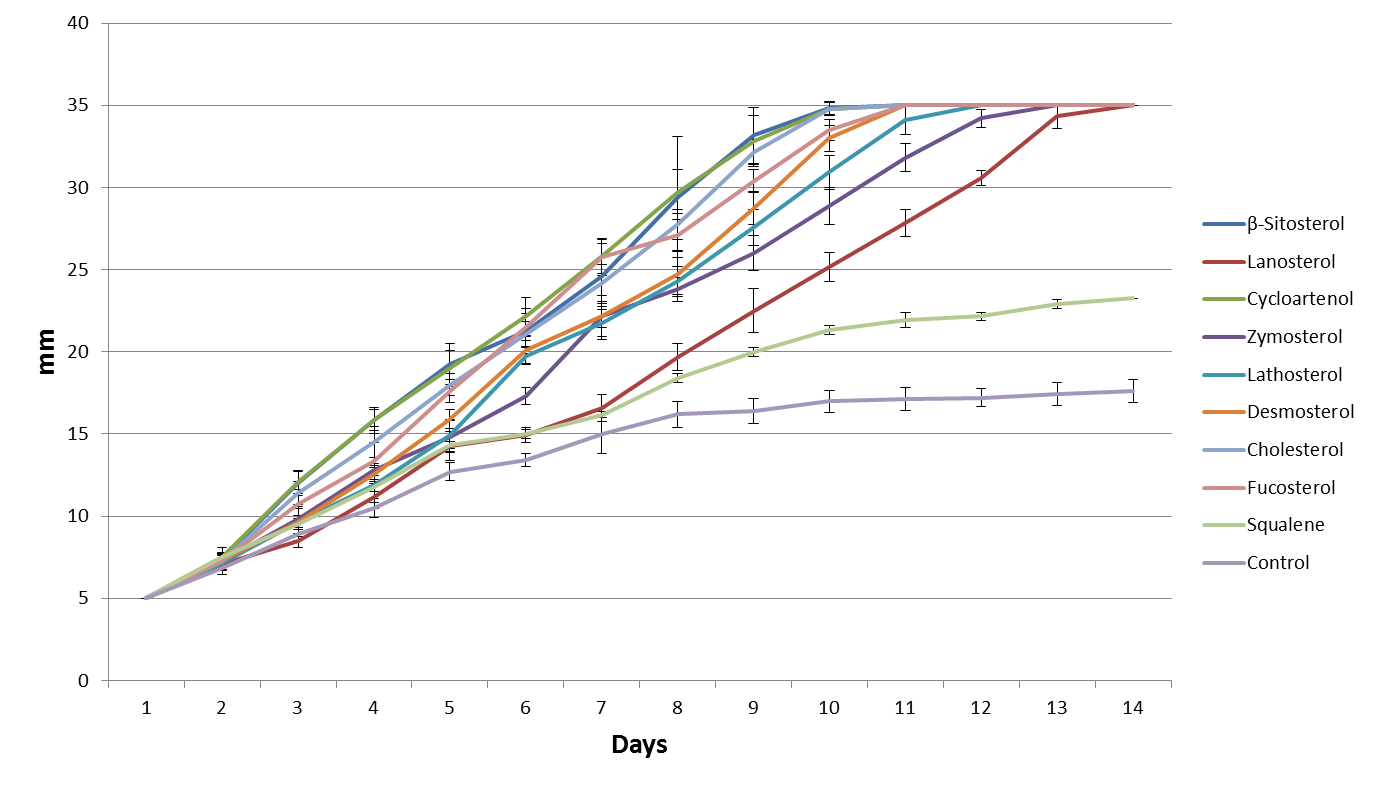


Figure C Growth of *Phytophthora infestans* on synthetic media supplemented with different sterols.

The colour key indicates the sterol or sterol precursor present in each experiment. The control culture contained no sterols and led to severely reduced growth. Growth on the sterol precursor squalene was also very poor. A 5 mm plug of excised mycelia was used to inoculate each growth medium. Six replicate experiments were performed in each case. Error bars represent one standard deviation from the mean.


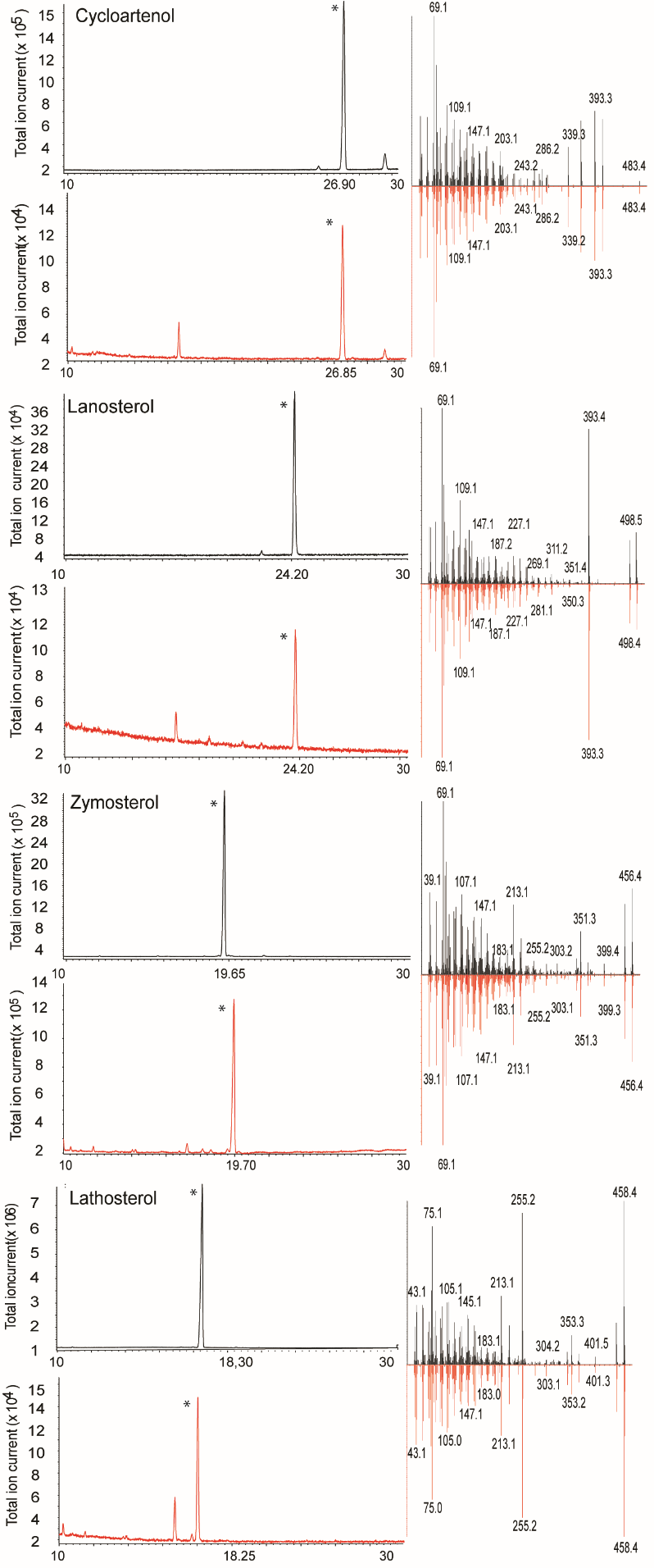

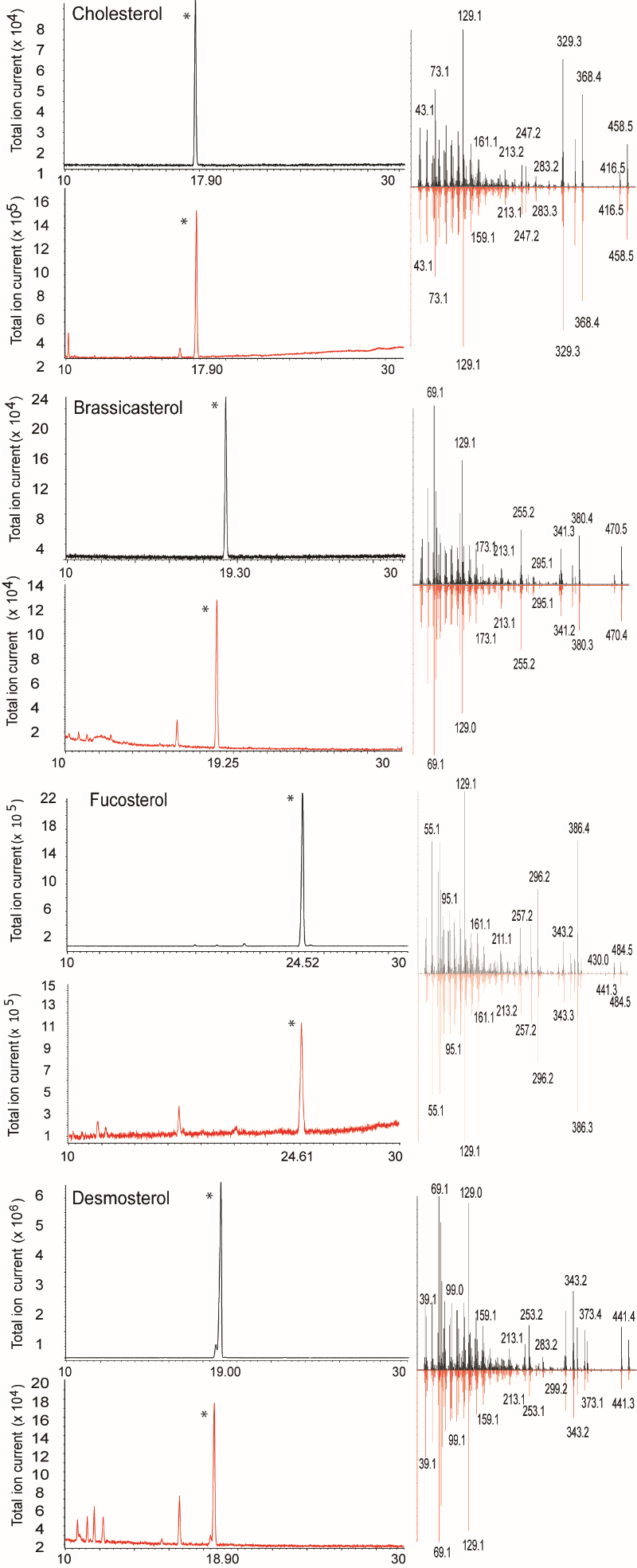

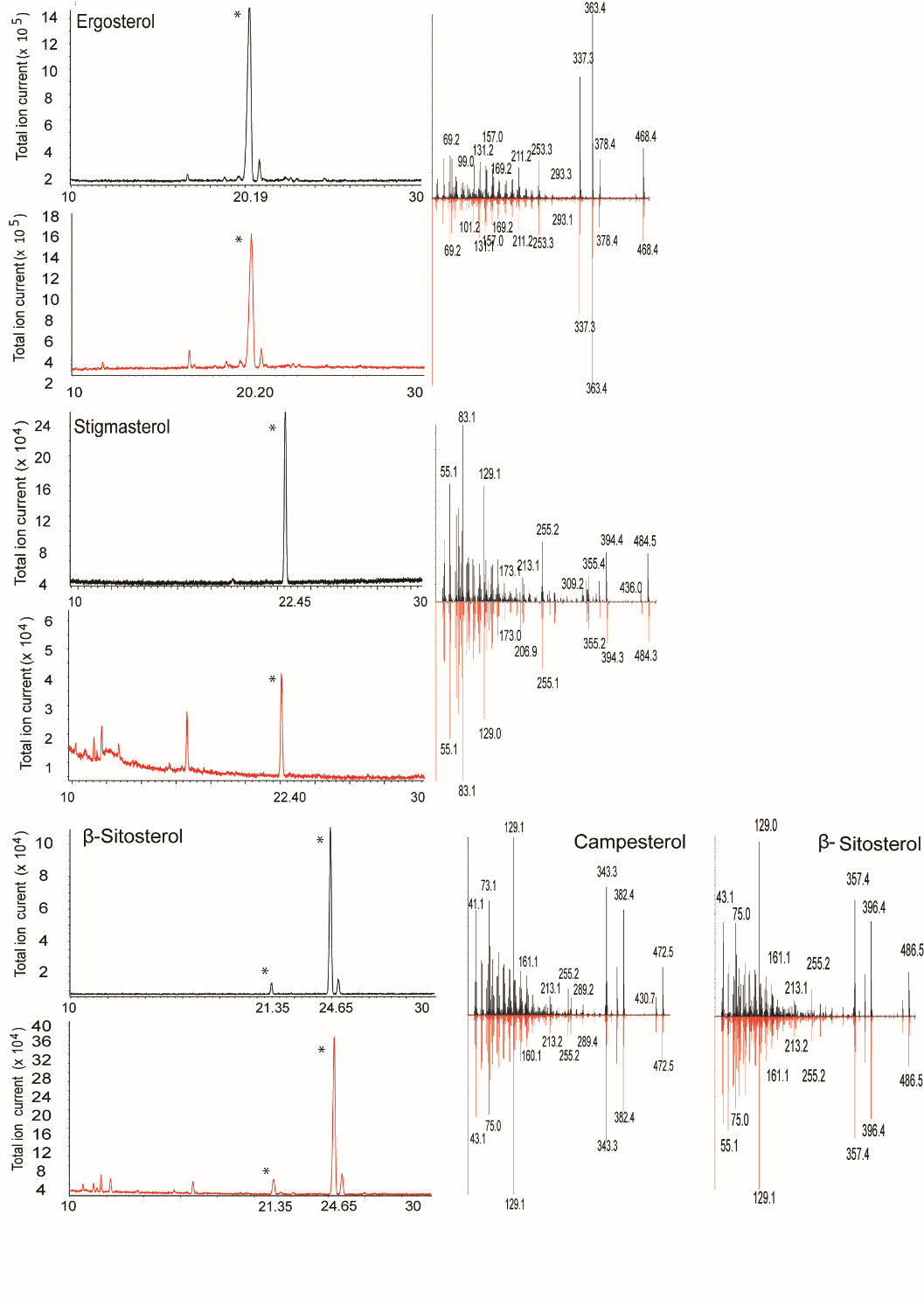


Figure D Various sterols fed to *P. infestans*, and subsequently extracted from mycelium, as investigated by Gas Chromatography coupled to Mass Spectrometry (GC-MS).

Each box represents a different sterol used in the feeding study, where the black spectra indicate sterol standards used for feeding and the red spectra indicate sterols extracted from 14-day-old *P. infestans* mycelia. The major peaks in the GC spectra are indicated in each case by an asterix (*), and the accompanying MS spectra show the important identifying m/z values in the fragmentation pattern. For β-sitosterol (top), two peaks can be seen in the gas chromatogram, a minor peak corresponding to campesterol, and a major peak corresponding to β-sitosterol (both confirmed by MS fragmentation analysis). Other small peaks could be observed, but fragmentation patterns showed that these did not correlate with any sterols, and are likely extract residues from the SPE column preparations.


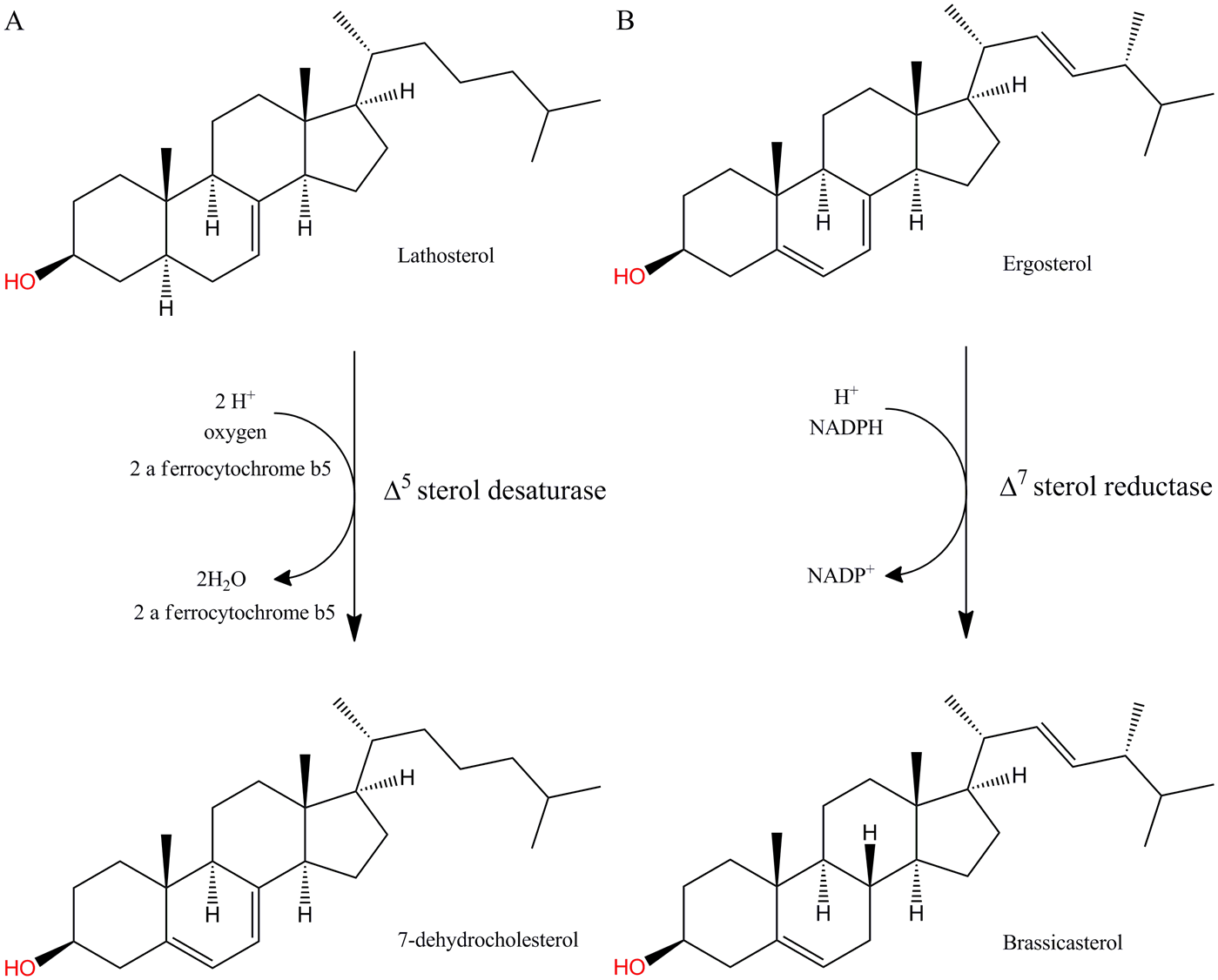


Figure E Putative sterol modifying activities of a Δ^5^ sterol desaturase and a Δ^7^ sterol reductase.

A: predicted conversion of lathosterol to 7-dehydrocholesterol by a Δ^5^ sterol desaturase. B: predicted conversion of ergosterol to brassicasterol by a Δ^7^ sterol reductase.
